# Congenital Chylothorax and Congenital Pulmonary Airway Malformation: Case Report and Literature Review

**DOI:** 10.1002/rcr2.70223

**Published:** 2025-06-16

**Authors:** Andréa L. Corso, Amanda M. Magdaleno, Carolina R. Cappellaro, Mateus M. Neves, Geórgia P. F. Oliveira, Iara R. S. Lucena, José C. Fraga

**Affiliations:** ^1^ Neonatology Service—Hospital de Clínicas of Porto Alegre (HCPA) Porto Alegre Brazil; ^2^ Department of Pediatrics, Faculty of Medicine Federal University of Rio Grande Do Sul (UFRGS) Porto Alegre Brazil; ^3^ Pediatric Thoracic and Airway Surgery Unit, Pediatric Surgery Service—Hospital de Clínicas of Porto Alegre (HCPA) Porto Alegre Brazil; ^4^ Radiology Service—Hospital de Clínicas of Porto Alegre (HCPA) Porto Alegre Brazil; ^5^ Department of Surgery, Faculty of Medicine Federal University of Rio Grande Do Sul (UFRGS) Porto Alegre Brazil

**Keywords:** congenital chylothorax, congenital pulmonary airway malformation, pleural effusion, pleurodesis, povidone‐iodine

## Abstract

The association of two rare but important congenital conditions—congenital chylothorax (CCT) and congenital pulmonary airway malformation (CPAM)—can be challenging to manage, especially in the absence of well‐established protocols. We report an association between CPAM and CCT in a newborn. After birth, CCT did not respond to conservative treatment, and at the time of CPAM resection, thoracic duct ligation and abrasive pleurodesis were also performed. Despite these interventions, the CCT persisted even with the subsequent administration of octreotide and propranolol. Finally, after 56 days, chemical pleurodesis with povidone‐iodine was performed. Chest tube drainage ceased, and the thoracic drain was removed 4 days later. Conservative treatment remains the first‐line approach for neonatal CCT. However, when CCT is associated with CPAM and fails to respond to conservative measures, thoracic duct ligation should be considered at the time of lung malformation resection. If these interventions remain ineffective, chemical pleurodesis is a viable therapeutic option.

## Graphical abstract

The association of two rare but important congenital conditions—congenital chylothorax (CCT) and congenital pulmonary airway malformation (CPAM)—can be challenging to manage, especially in the absence of well‐established protocols. We report our experience and management of an association between CPAM and CCT in a newborn.

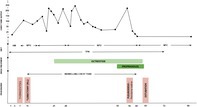

## 1 Introduction

Congenital pulmonary airway malformation (CPAM), a rare condition with an incidence of 1 in 10,000–30,000 births, is nevertheless the most common congenital lung anomaly, accounting for approximately 95% of pulmonary cystic malformations. CPAM can occur at different stages of lung development, leading to abnormal bronchial morphogenesis [1], and is often diagnosed during the prenatal period. It usually affects one lobe (especially the lower lobe), with multilobar or bilateral disease occurring less frequently [2]. Most CPAM cases are sporadic, without association with maternal factors or genetic predisposition [3].

In turn, another rare condition—congenital chylothorax (CCT)—is the most common cause of pleural effusion in newborns. CCT is associated with a mortality rate of approximately 20% [4]. It is often linked to chromosomal abnormalities, congenital heart defects, intrauterine infection, or lymphatic malformations, but it can also present as an isolated condition [5]. Chylothorax results from the accumulation of lymphatic fluid in the pleural space due to obstruction or leakage of lymphatic vessels or the thoracic duct [6]. Diagnosis is based on the milky appearance of the pleural fluid, triglyceride levels > 110 mg/dL, and a cell count > 1000 cells/mL with a lymphocyte percentage > 80% [7].

The co‐occurrence of CCT and CPAM is extremely rare [8], and although well‐established treatments are available for each condition independently, the management of the combined diseases is still undefined and controversial.

## 2 Case Report

The foetus of a healthy 20‐year‐old woman was diagnosed with a lung lesion via ultrasound and was subsequently followed by the foetal medicine service at a quaternary hospital. At 31 weeks of gestational age, the foetus developed pleural and pericardial effusion, prompting a caesarean section. The male newborn, with a birth weight of 2000 g, required continuous positive airway pressure for resuscitation and stabilisation in the delivery room. He was then transferred to the neonatal intensive care unit, where he required intubation and mechanical ventilation due to ventilatory insufficiency within the first hour of life. A chest X‐ray revealed a solid mass in the right hemithorax. An echocardiogram showed no signs of external compression and minimal pericardial effusion. Thoracic ultrasound showed a heterogeneous image in the middle/lower third of the right lung, with lobulated contours, internal cystic areas, and echogenic foci, consistent with a diagnosis of CPAM. Moderate ipsilateral pleural effusion was also detected. The patient developed clinical signs of pulmonary hypertension and required high‐frequency ventilation. Total parenteral nutrition (TPN) was initiated on the first day of life (DOL).

**FIGURE 1:**
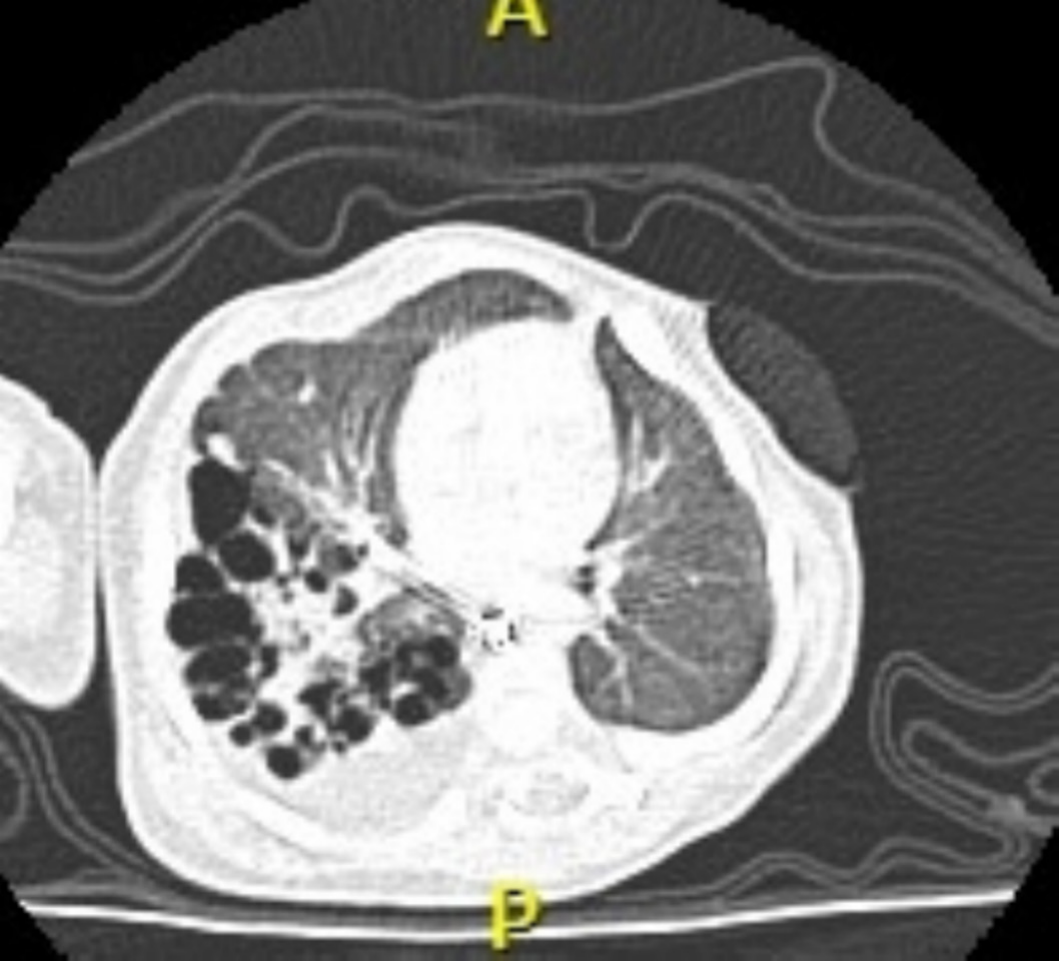
Computed tomography (CT) of the chest illustrating multiple cystic malformations in the lung parenchyma of the superior, lateral, and posterior segments of the right lower lobe, consistent with CPAM. Moderate pleural effusion was also noted on the right side.

On the 5th DOL, there was clinical improvement, and the newborn was able to receive breast milk via an orogastric tube. However, pleural effusion increased on the 7th DOL. Thoracentesis was performed with removal of 100 mL of citrine fluid with 95% lymphocytes and 1417 mg/dL triglycerides, consistent with CCT. Conservative treatment was initiated with no food by mouth and maintenance of TPN. On the 8th DOL, a chest computed tomography (CT) scan (Figure 1) revealed multiple thin‐walled cystic formations of varying sizes (up to 1 cm in diameter) within lung parenchyma of the superior, lateral, and posterior segments of the right lower lobe (RLL), consistent with type 2 CPAM. Moderate free‐appearing posterior pleural effusion was also noted on the right side.

With the patient clinically stable and still on mechanical ventilation, a right thoracotomy was performed on the 11th DOL, with RLL lobectomy, thoracic duct ligation at the right hemithorax entry, abrasive pleurodesis by pleural scarification, and placement of a chest drain on the right side. Because visualisation of the thoracic duct was prevented by the newborn's fasting status, a mass supradiaphragmatic ligation of all tissue between the aorta and azygos vein was performed using non‐absorbable suture. The pathology analysis confirmed type 2 CPAM. Genetic evaluation revealed a 46, XY karyotype with no other associated malformations.

**FIGURE 2:**
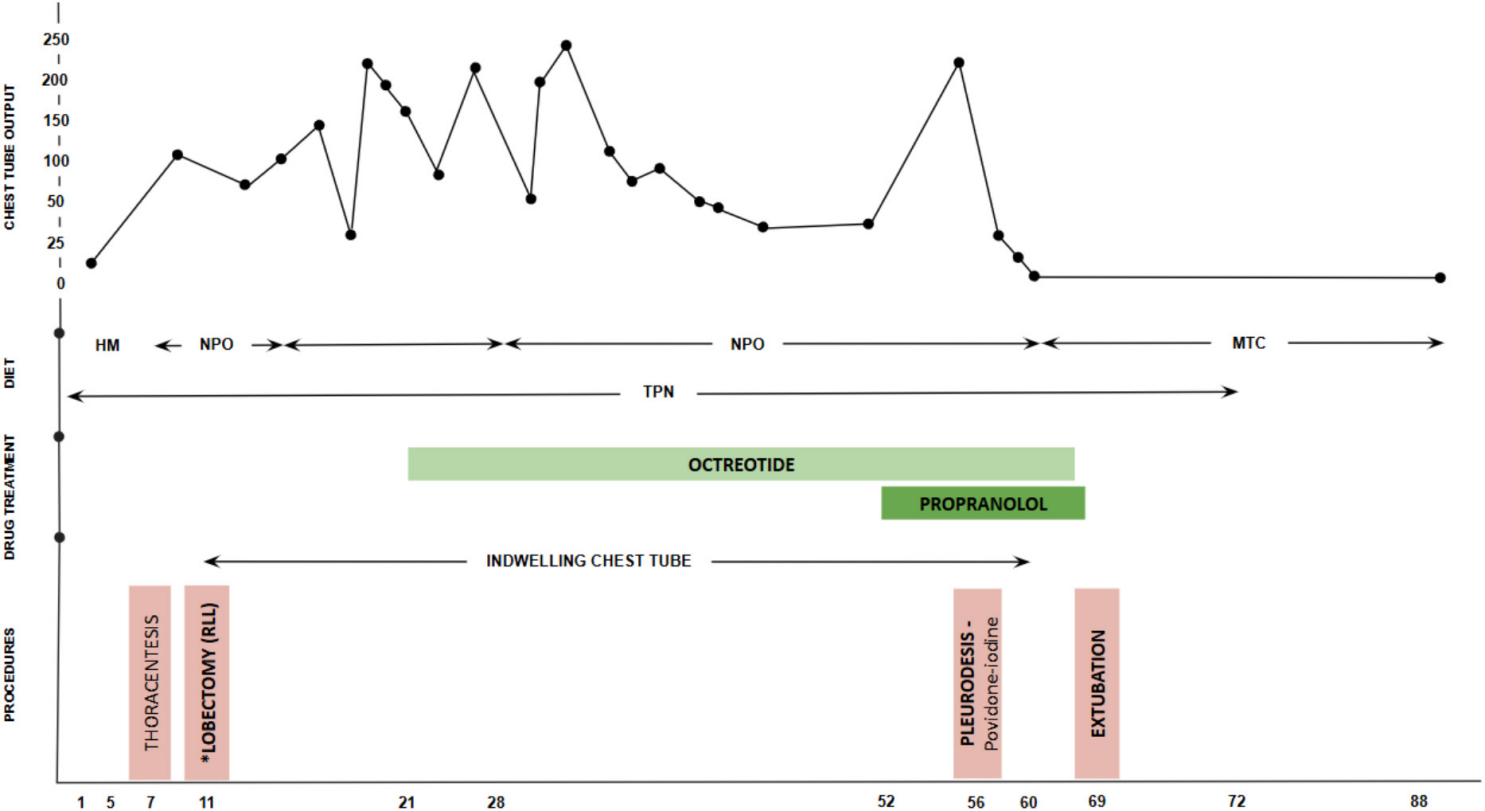
Chest tube output, drug treatment and procedures according to days of life. HM: human milk; MTC: medium‐chain triglyceride; NPO: no food by mouth; TPN: total parenteral nutrition. *Lobectomy right lower lobe (RLL) + thoracic duct ligation + abrasive pleurodesis.

On the 21st DOL, significant drainage persisted through the chest drain, and intravenous octreotide was administered at an initial dose of 1 μg/kg/h; the dose was gradually increased to 12 μg/kg/h. Intravenous immunoglobulin (IgG) 400 mg/kg was given on the 22nd DOL due to IgG deficiency. Albumin replacement was performed on the 26th DOL due to hypoalbuminemia. At 52 DOL, propranolol 0.3 mg/kg/day was initiated via an orogastric tube due to persistent pleural effusion, and was increased to 0.7 mg/kg/day (Figure 2).

**FIGURE 3:**
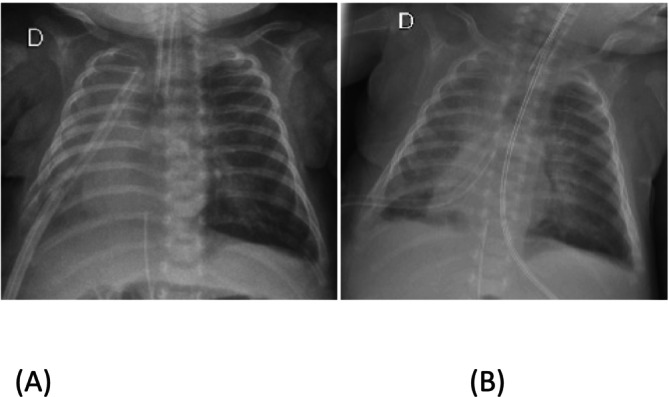
Chest X‐ray before (A) and after treatment with pleurodesis using povidone‐iodine (B).

Given the increased pleural effusion (Figure 3A), on the 56th DOL, chemical pleurodesis was performed by instilling 5 mL (2 mL/kg) of 4% povidone‐iodine into the pleural space through the thoracic drain. The chest drain was clamped for 5 h, then reopened and left on water seal. The procedure was uneventful. Drainage gradually decreased and ceased completely on the 60th DOL (Figure 3B).

A medium‐chain triglyceride (MCT) diet was started. The chest drain was removed and the enteral diet was progressed with good tolerance. On the 69th DOL, the patient was extubated and began receiving full enteral nutrition. Parenteral nutrition was stopped (Figure 2). The patient was discharged from the hospital in room air, receiving an MCT diet via an orogastric tube. A brain MRI performed at term‐equivalent age was normal at discharge. Full oral feeding was achieved by the 4th month of life.

In the follow‐up after discharge, the patient had one hospitalisation for viral bronchiolitis at 7 months of age, requiring only oxygen support. Currently, at 12 months, imaging studies show the absence of right pleural effusion and expansion of the right upper lobe, but persistent hyperinflation of the left lung.

## 3 Discussion

Congenital lung malformations are rare anomalies of lung development. CPAM, the most common of them, is characterised by excessive growth of terminal bronchioles at the expense of alveoli. Type 2 CPAM, diagnosed in our patient, represents about 15%–30% of all cases and is believed to originate in the bronchioles [9].

In the present case, surgical management of CPAM was performed during the neonatal period due to the patient's ventilatory insufficiency from birth, including the need for invasive respiratory support. Open surgery via thoracotomy was indicated because the patient was on mechanical ventilation prior to surgery, and would not tolerate video‐assisted thoracoscopic surgery. While the thoracoscopic approach is superior to traditional open surgery in several aspects [10, 11, 12], it was not an option for our patient.

CCT is a potentially serious and life‐threatening [7] condition resulting from an abnormality in the lymphatic vessels. The presence of CPAM likely interfered with the formation of intrathoracic lymphatics, causing their rupture with subsequent accumulation of lymph in the pleural space.

The literature reports that 14% of patients with congenital lung lesions have associated malformations [13]. Although we did not detect syndromes or cardiac malformations in our patient, CCT was present on the same side as the lung lesion. This association between CPAM and CCT has been described only once in the literature, but no information on clinical or surgical management was provided [8]. Conservative management is the first‐line therapy for CCT [7, 8, 14, 15]; in our patient, CCT did not respond to these treatments. Therefore, thoracic duct ligation and abrasive pleurodesis were performed during the resection of the lung lesion.

Octreotide is a somatostatin analog used as a therapeutic option for the treatment of both congenital and acquired chylothorax [7, 14, 15]. Octreotide acts on splanchnic vessels by reducing intestinal blood flow, gastrointestinal motility, and fat absorption, thereby reducing lymph flow and chylous accumulation in the thorax [16]. Octreotide was used in our patient but did not cease lymph drainage through the chest tube. Our patient also received propranolol to reduce pleural drainage, as propranolol has been reported to be effective for some congenital lymphatic vessel anomalies such as CCT. However, drainage persisted even after the use of propranolol [17].

Our patient presented with factors associated with higher morbidity and mortality, such as prematurity and persistent chylous drainage [18], which led to an increased risk of malnutrition, infection, and death. Monitoring and managing fluid, electrolyte, and nutritional imbalances were of utmost importance, as was the replacement of volume, proteins, and immunoglobulins. Serum albumin was maintained above 2.5 g/dL. Constant vigilance and concern for late‐onset sepsis were also crucial in managing this neonate.

Chylothorax resistant to conservative, medical therapy and drainage is usually treated by surgery and chemical pleurodesis [4]. Because our patient required thoracotomy for CPAM resection, surgical treatment of CCT was performed simultaneously with thoracic duct ligation and abrasive pleurodesis. Since there was no improvement in chylothorax postoperatively, intravenous octreotide was administered, followed by propranolol, but without ceasing pleural drainage. With no response to conservative, pharmacological, and surgical treatments, chemical pleurodesis with povidone‐iodine was chosen.

Pleurodesis agents include talc, minocycline, OK‐432, bleomycin, and povidone‐iodine [14, 19, 20]. Pleurodesis with OK‐432 is mostly used in the prenatal treatment of foetuses with chylothorax and hydrops fetalis [21]. Povidone‐iodine pleurodesis in neonates was first reported in 2003 by Brissaud et al. [22], who treated four newborns with CCT. Since that first report, several other cases have been described in the literature [23, 24, 25, 26]. However, the use of povidone‐iodine pleurodesis in neonates with CCT is still fairly controversial due to concerns about complications such as renal failure, hyperthyroidism, allergic reactions, and cardiorespiratory reactions [27]. The pleurodesis performed in our patient was uneventful, and lymphatic drainage through the chest tube ceased 4 days after administration of povidone‐iodine, with no evidence of short or long‐term adverse effects. Since a protocol for povidone‐iodine pleurodesis in neonates is not available, there is significant variability in how the procedure is performed [22, 27, 28]. We chose to clamp the drain for 5 h after application to allow the povidone‐iodine to spread throughout the pleural space. After the use of intrapleural povidone‐iodine, it is important to monitor thyroid and renal function before and after treatment; in our patient, the tests were normal during hospitalisation and on long‐term follow‐up.

The association of CPAM with CCT in a newborn is very rare. Even after surgical resection of the CPAM and surgical treatment of chylothorax by concomitant thoracic duct ligation and pleural abrasive pleurodesis, intrapleural lymphatic drainage did not improve in our patient. Attempts to use octreotide and propranolol were also ineffective in resolving the chylothorax. We believe that in newborn patients with CPAM associated with CCT, in whom resection of the pulmonary malformation and surgical treatment of the CCT are not effective, early chemical pleurodesis should be performed. Although there is still controversy about the use of pleurodesis with povidone‐iodine in newborns, it proved to be effective and safe for the treatment of our patient with chylothorax refractory to conservative and surgical measures.

## Author Contributions

José C. Fraga and Andréa L. Corso conceived and designed the study. José C. Fraga, Andréa L. Corso, Amanda M. Magdaleno, Carolina R. Cappellaro, Georgia P. F. Oliveira, and Mateus M. Neves cared for the patient during hospitalisation. Iara R. S. Lucena performed and interpreted all imaging exams during hospitalisation and guided pleural puncture procedures. José C. Fraga, Andréa L. Corso, Amanda M. Magdaleno, and Carolina R. Cappellaro collected and interpreted all relevant clinical data. José C. Fraga, Andréa L. Corso, Amanda M. Magdaleno, Carolina R. Cappellaro, Georgia P. F. Oliveira, Mateus M. Neves, and Iara R. S. Lucena prepared the manuscript. José C. Fraga, Andréa L. Corso, Amanda M. Magdaleno, Carolina R. Cappellaro, Georgia P. F. Oliveira, Mateus M. Neves, and Iara R. S. Lucena confirm the authenticity of all the raw data in this study. All authors read and approved the final manuscript.

## Ethics Statement

The authors declare that written informed consent was obtained for the publication of this manuscript and accompanying images using the consent form provided by the journal. This publication was approved by the ethics committee (Hospital de Clínicas de Porto Alegre [HCPA], Brazil, number 2024–0440‐CAAE: 83905524.6.0000.5327).

## Conflicts of Interest

The authors declare no conflicts of interest.

## Data Availability

The data that support the findings of this study are available on request from the corresponding author. The data are not publicly available due to privacy or ethical restrictions.
